# Comparative physiology and biomimetics in metabolic and environmental health: what can we learn from extreme animal phenotypes?

**DOI:** 10.1007/s00125-025-06611-3

**Published:** 2025-11-20

**Authors:** Peter Stenvinkel, Peter Kotanko, Johanna Painer-Gigler, Paul G. Shiels, Pieter Evenepoel, Leon Schurgers, Barbara Natterson-Horowitz, Szilvia Kalogeropoulu, Joshua Schiffman, Richard J. Johnson

**Affiliations:** 1 Division of Renal Medicine, Department of Clinical Science, Intervention and Technology, Karolinska Institutet, Stockholm, Sweden; 2 Renal Research Institute, New York, NY USA; 3 Icahn School of Medicine at Mount Sinai, New York, NY USA; 4 Research Institute of Wildlife Ecology, Department of Interdisciplinary Life Sciences, University of Veterinary Medicine, Vienna, Austria; 5 Glasgow Geroscience Group, School of Molecular Biosciences, MVLS, University of Glasgow, Glasgow, UK; 6 Laboratory of Nephrology, KU Leuven Department of Microbiology, Immunology and Transplantation, KU Leuven, Leuven, Belgium; 7 Department of Biochemistry, Cardiovascular Research Institute Maastricht, Maastricht University, Maastricht, the Netherlands; 8 Division of Cardiology, UCLA School of Medicine, Los Angeles, CA USA; 9 Department of Pediatrics, Huntsman Cancer Institute, University of Utah, Salt Lake City, UT USA; 10 Peel Therapeutics, Inc., Salt Lake City, UT USA; 11 Department of Medicine, University of Colorado Anschutz Medical Campus, Aurora, CO USA

**Keywords:** Biomimetics, Cardiovascular disease, Climate change, Comparative physiology, Exposome, Hibernation, Insulin resistance, Longevity, Metabolic adaptation, Nrf2, Planetary health, Review

## Abstract

This review explores the remarkable metabolic adaptations of species that thrive in extreme environments, providing insights into their resilience, flexibility and disease resistance. Species such as hibernating brown bears, migratory birds, cavefish, Greenland sharks and naked mole rats exhibit unique metabolic traits that challenge conventional paradigms of metabolic regulation. These adaptations, including resistance to hypoxia and metabolic ageing, offer potential solutions to human metabolic disorders, including obesity, type 2 diabetes and CVD. Insights from comparative physiology, particularly the mechanisms by which animals cope with food scarcity, extreme temperatures and hypoxia, could help identify novel therapeutic targets for advancing human health. For example, hibernation can serve as a model for understanding metabolic diseases, providing insights into reversible insulin resistance and energy homeostasis. This review also highlights the impact of environmental stressors, including climate change, on these species, which may jeopardise their survival despite their resilience. Accelerating anthropogenic environmental change threatens even the most resilient animal species. We call for a holistic approach to conservation and environmental protection to preserve these species and the valuable lessons they offer for managing our metabolic health.

**Graphical Abstract:**

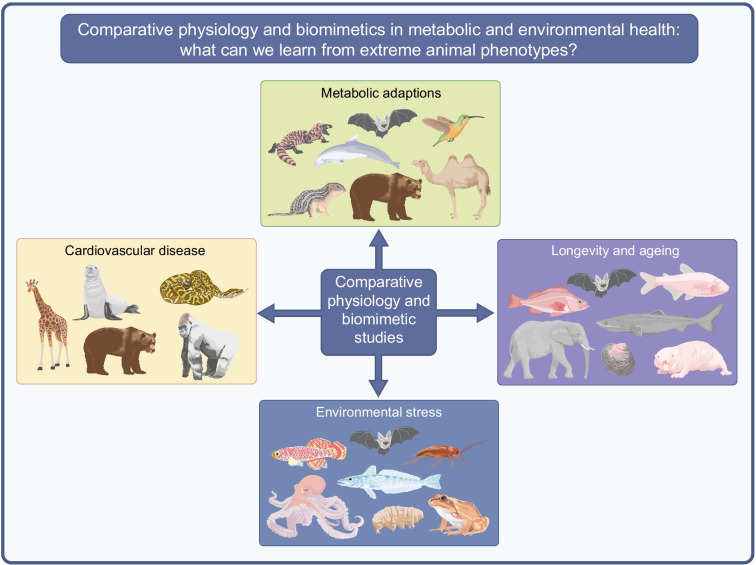

**Supplementary Information:**

The online version contains a slideset of the figures for download available at 10.1007/s00125-025-06611-3.

## Insights from comparative physiology for human health and disease

Metabolic disorders, including obesity, type 2 diabetes and CVD, are major health challenges in the Anthropocene, the current geological period during which human activity has been the dominant influence on the climate and the environment. These metabolic disorders contribute to epidemics of non-communicable diseases, which lead to premature biological ageing and significant disparities in healthspan and lifespan [1]. Research has provided extensive insights into human metabolic physiology; however, gaps remain in our understanding of adaptative mechanisms, which can inspire novel strategies for the prevention and treatment of human disease. Other promising, yet underexplored avenues are comparative physiology and biomimetics, the study of how different organisms adapt their metabolism to thrive in extreme environments [2].

Across the animal kingdom, numerous species have evolved remarkable metabolic traits to cope with harsh or fluctuating exposomes [3], including prolonged fasting, dehydration, hypoxia, cold exposure or nutrient scarcity. Lessons gleaned from biomimetic investigations of diverse wildlife—from hibernating brown bears (*Ursus arctos*) and hummingbirds (Trochilidae), to elephants (*Loxodonta africana*) and bats (Chiroptera)*—*provide a cornucopia of natural insights for combatting non-communicable diseases and environmental threats [2], showcasing the untapped potential residing within natural systems. Extreme phenotypes challenge traditional paradigms of metabolic regulation and provide unique biological insights into resilience, flexibility and disease resistance. These adaptive phenotypes have become even more pronounced under the rapidly changing environmental conditions [4].

This review explores the diverse physiological adaptations of animals with extreme metabolic phenotypes and considers how these strategies may inform our understanding of human metabolic health. Learning from nature may also provide opportunities for environmental management and solutions to the most challenging issues that threaten the future of the planet, such as climate change (including global warming), water shortages, exposure to plastics and air pollution [3].

## Metabolic adaptations in animals: insights into insulin resistance, obesity and survival

Periodic food shortages are a major challenge faced by organisms in their natural habitats. In nature, obesity and insulin resistance function as evolutionary adaptations. Many animals increase their fat stores to prepare for food scarcity, pregnancy or lactation and support their energy needs. Desert rodents store fat in viscera and tails, while larger species (e.g. camels [*Camelus bactrianus*], oryx [Bovidae]) concentrate fat in humps or visceral depots, minimising subcutaneous fat to maximise heat dissipation [5]. Marine mammals such as walruses (*Odobenus rosmarus*) and pinnipeds (Phocidae) accumulate subcutaneous fat that aids with insulation and buoyancy in cold waters, but which generates metabolic water and energy when fasting [6]. Other animals, such as orangutans (*Pongo*) and lemurs (Lemuriformes)*,* increase their fat stores before dry seasons, while species living in extreme seasonal conditions (e.g. emperor penguins, bears, dormice, marmots) build fat reserves before winter [7].

Transient insulin resistance emerges in many species as a survival mechanism, with avoidance of diabetes and its complications. Such adaptive insulin resistance typically occurs alongside fat deposition in preparation for hibernation, long-distance migration or nesting [8]. Reduced glucose uptake in skeletal muscle conserves energy while maintaining elevated blood glucose to fuel insulin-independent regions in the brain. Meanwhile, the decreased glucose uptake that occurs in insulin-dependent brain areas triggers foraging behaviour. Although insulin resistance is commonly associated with food intake, weight gain and fat storage, it may also occur during fasting, exemplified by the hibernating brown bear [9].

Aquatic organisms can also provide valuable insights into the prevention of complications of insulin resistance. Bottlenose dolphins (*Tursiops truncatus*)*, *for example, shift between insulin resistance during fasting and insulin sensitivity when fed [10]. Their ability to remain healthy despite prolonged postprandial hyperglycaemia and hyperinsulinaemia supports their utility as a model for diabetes research [11]. They can maintain brain glucose levels during fasting, or long dives, underscoring their ability to engender a metabolic switch, a vital feature for energy management on a low-carbohydrate, variable diet. Another mammal, the northern elephant seal (*Mirounga angustirostris*)*,* exhibits flexible insulin resistance during prolonged fasting associated with diving, breeding, moulting and post-weaning. During these periods, energy is largely derived from fat oxidation, despite marked insulin resistance and fasting hyperglycaemia, while the expression of the cytoprotective transcription factor nuclear factor erythroid 2-related factor 2 (Nrf2) is upregulated [12]. Pups can fast for up to 12 weeks during rapid growth, relying entirely on metabolic water from fat oxidation [13]. Furthermore, elephant seal muscle mass is protected from wasting, even after weeks of immobility and fasting [14]. Despite sustained fat oxidation, ketosis appears to be absent in elephant seals, making them a model for studying ketoacidosis in humans [15].

The blind cavefish (*Astyanax mexicanus*)*,* which includes both surface and cave ecotypes, endures long periods of nutrient deprivation and must rely on external energy inputs such as seasonal floods for survival. To assist survival, cave-adapted populations exhibit more severe insulin resistance than river-dwelling populations. Despite this insulin resistance, they have lifespans that are comparable to those of their surface-dwelling counterparts and do not accumulate AGEs, which are linked to diabetes-related pathologies. Thus, cavefish have evolved compensatory mechanisms that protect them from the harmful consequences of insulin resistance and impaired blood glucose regulation [16]. It was recently reported that metabolic shifts, particularly in mitochondrial function, may contribute to the cavefish’s extended longevity [17].

Differential adaptations to nutritional stressors can also be found among avian species, which provide unique insights into disease and adverse human lifestyle factors. Among birds, fasting glucose levels are highest in carnivores (from gluconeogenesis) and lowest in nectarivores/frugivores, suggesting that the latter have evolved adaptations to mitigate hyperglycaemia after feeds [18]. Nevertheless, hummingbirds consume fructose- and glucose-rich nectar daily, resulting in severe postprandial hyperglycaemia (>28 mmmol/l) associated with insulin resistance and fatty liver [19, 20]. Despite this marked hyperglycaemia, they appear to be protected from diabetic or hepatic complications. The hyperglycaemia quickly resolves due to their high metabolic rate (e.g. heart rates up to 1200 beats per minute [bpm] during flight), a unique biochemical protective system (glycation resistance, upregulated antioxidant system) and genetic advantages [21, 22]. Studies of flying mammals, in particular the neotropical bats, which exhibit unusually high expression levels of the glucose transporter gene, can provide additional insights into the mechanisms underlying dietary diversification and sugar assimilation [23].

Another exposome factor that many species have adapted to is water deprivation. This is a life-threatening condition that triggers a protective physiological response linking osmolyte retention to enhanced thirst. While this mechanism safeguards against short-term dehydration, it is insufficient to sustain long-term water balance. A study on thirteen-lined ground squirrels (*Ictidomys tridecemlineatus*) has demonstrated that evolutionarily conserved brain regions regulating fluid homeostasis in mammals possess a remarkable capacity to support long-term survival without water [24].

**Fig. 1:**
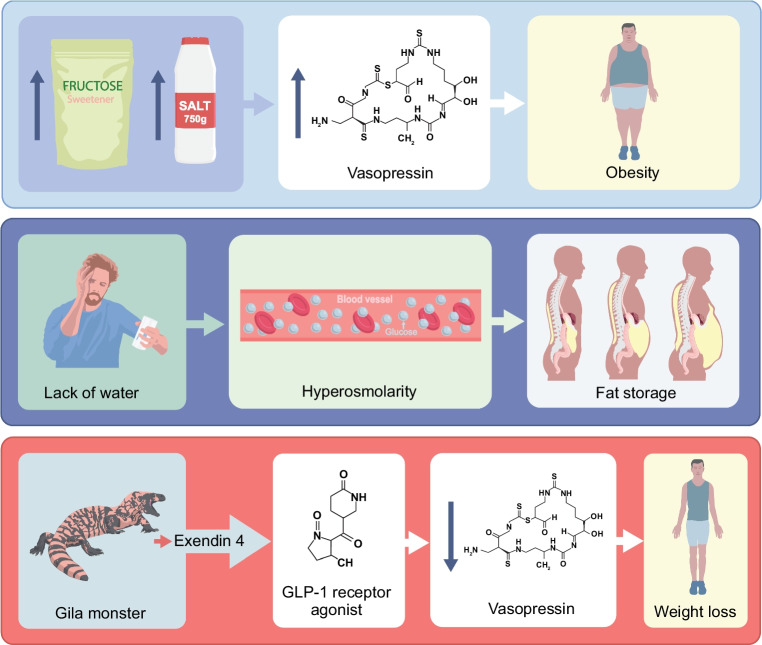
Insights from nature elucidate the interplay between water metabolism, vasopressin and obesity. Dietary salt, fructose intake and inadequate access to clean water stimulate vasopressin secretion and induce hyperosmolarity, thereby promoting adipogenesis and fat accumulation. The discovery of exendin-4*,* a glucagon-like peptide-1 (GLP-1) analogue isolated from the Gila monster has led to the development of GLP-1 receptor agonists, which exert weight-reducing effects partly through the suppression of vasopressin signalling. This figure is available as part of a downloadable slideset

Fat stores are not just an energy reserve but also generate water (~1.1 ml/g). Although net water generation is constrained by respiratory water vapour loss associated with increased oxygen demand, desert, marine and hibernating mammals have evolved mechanisms to reduce evaporative water loss. Recognising that fat serves as a source of metabolic water has helped uncover vasopressin’s role in promoting obesity as part of a water conservation strategy, mediated through activation of the vasopressin 1b receptor, which increases ACTH, glucagon and cortisol levels (Fig. 1) [25, 26]. Mild dehydration is increasingly recognised in metabolic disorders [27], manifesting as mild hyperosmolarity and elevated levels of serum copeptin, a vasopressin precursor [28–30].

Desert animals have also developed ingenious metabolic mechanisms to survive dehydration or famine. Bactrian camels, for example, can survive for 2–4 weeks without food or water. Camels reduce their metabolic rate and become insulin resistant, hyperglycaemic and hypercholesterolaemic during famine, but revert to insulin sensitivity when feeding. Despite their large fat stores and high blood lipid levels, camels do not experience obesity-related insulin resistance. Interestingly, camel milk not only alleviates hyperglycaemia and improves lipid profiles, insulin secretion and insulin sensitivity in individuals with diabetes [31], but also stimulates the transcription factor Nrf2, which controls the expression of >200 cytoprotective genes involved in antioxidant defence, inflammation regulation and mitochondrial biogenesis [32].

Biomimetic studies also emphasise how insights from nature can provide clues to the treatment of metabolic disorders (Fig. 1). Studies of the Gila monster (*Heloderma suspectum*), a venomous lizard native to the southwestern USA, led to the development of glucagon-like peptide-1 (GLP-1) receptor agonists. Their saliva contains exendin-4, a peptide with GLP-1 activity that has been modified to generate exenatide, the first GLP-1 receptor agonist [31]. Although GLP-1 receptor agonists enhance insulin secretion, this mechanism is unlikely to fully account for its remarkable ability to treat obesity. Thus, it is of interest that GLP-1 receptor agonists inhibit vasopressin and glucagon, which drive fat storage [33]. Similarly, increased water intake suppresses vasopressin secretion and ameliorates features of the metabolic syndrome in animals, with comparable trends reported in humans [34].

## Ageing and resilience: how long-lived species adapt to environmental stress

**Fig. 2:**
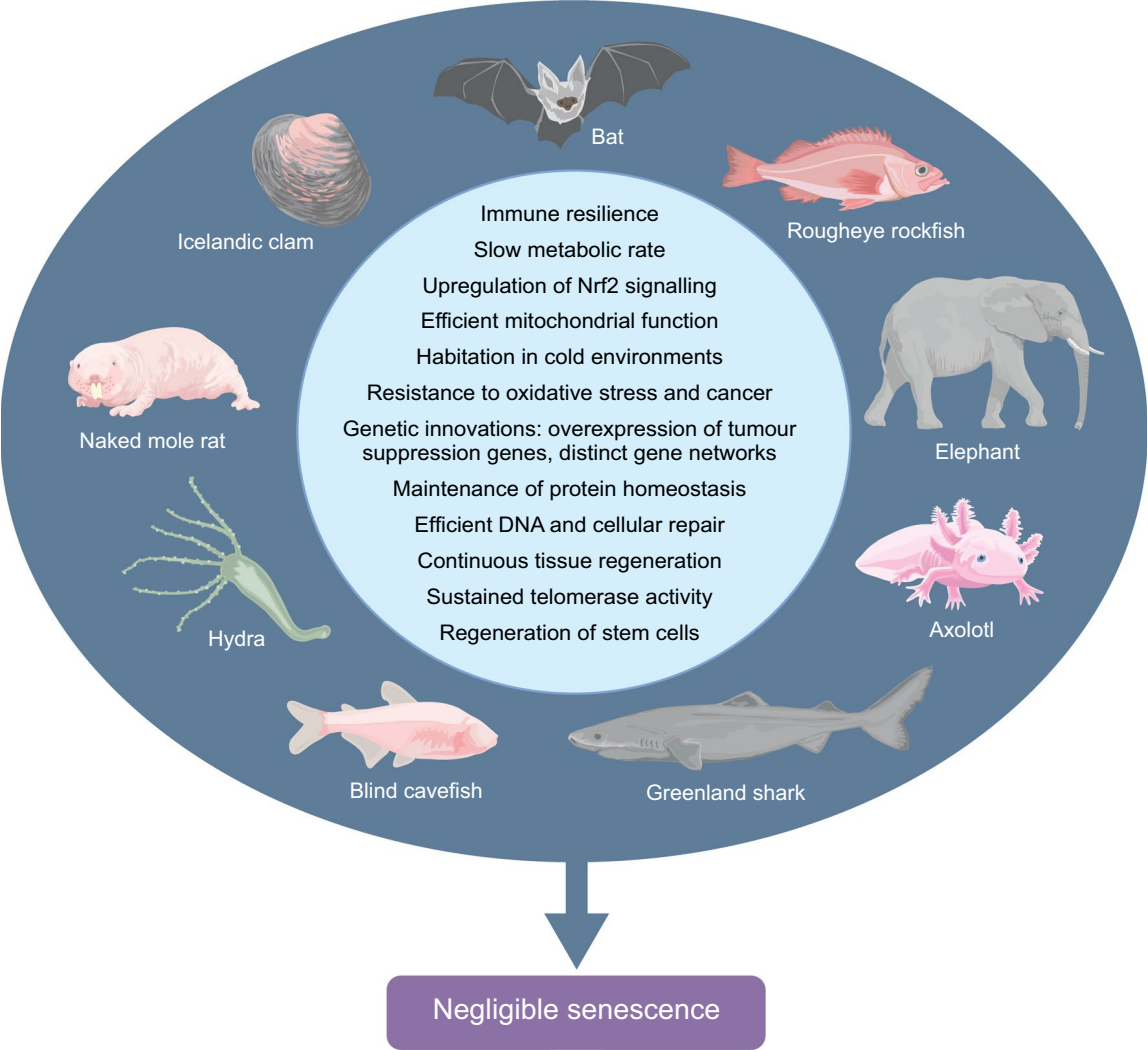
Long-lived organisms with negligible senescence provide valuable insights for geroscience. Bats demonstrate exceptional immune resilience and enhanced DNA repair capacity, contributing to their extended lifespan despite high metabolic activity. Naked mole rats exhibit upregulated Nrf2 signalling, efficient DNA repair and efficient mitochondrial function, all of which confer remarkable resistance to oxidative stress and cancer. Icelandic clams’ extreme longevity is linked to minimisng metabolic and oxidative stress, maintaining exceptional cellular homeostasis and inhabiting a cold, low-energy environment that favours maintenance over rapid growth and reproduction. Rougheye rockfish have two distinct gene networks related to insulin signalling and flavonoid metabolism that have been implicated in its exceptional lifespan. Greenland shark possess the lowest metabolic rate among vertebrates and exhibit enhanced DNA repair capacity. Their tissues contain high levels of trimethylamine *N*-oxide (TMAO)**,** which stabilises proteins and mitigates the destabilising effects of urea and cold temperatures. The axolotl maintains enhanced proteostasis, efficient DNA repair and resistance to oxidative stress, coupled with stable telomere length and low cellular senescence throughout life. The extended lifespan of blind cavefish is attributed to their low metabolic rate, enhanced stress resistance, and physiological adaptations that mitigate inflammation and oxidative damage, making them a valuable model for ageing research. Elephants are interesting for ageing research because they combine large body size, long lifespan and remarkable resistance to cancer and age-related disease, a combination that seems paradoxical from an evolutionary and biological perspective. Finally, the hydra represents a unique case of apparent biological immortality, enabled by perpetual stem cell renewal, sustained telomerase activity, continuous tissue regeneration, and exceptional genomic and proteomic maintenance. Collectively, these organisms demonstrate that longevity can evolve through diverse yet convergent strategies, including metabolic suppression, efficient cellular repair, oxidative stress resistance and stem cell maintenance. This figure is available as part of a downloadable slideset

As well as humans, other smaller and bigger animals also demonstrate remarkable resilience and longevity, providing valuable insights into how biology can adapt to extreme or evolving exposomes (Fig. 2) [35]. Long-lived species include naked mole rats (*Heterocephalus glaber*)*,* Greenland sharks (*Somniosus microcephalus*), elephants, bats, Icelandic clams (*Arctica islandica*)*,* axolotls (*Ambystoma mexicanum*)*,* hydra (*Hydra vulgaris*) and rougheye rockfish (*Sebastes aleutianus*) [36]. Common features observed in animals with negligible senescence include habitation in cold environments, upregulated Nrf2, efficient DNA repair, coherent mitochondrial function and maintenance of protein homeostasis [36]. Of special interest is the hydra, a tiny freshwater polyp that does not age due to continuous regeneration of stem cells [37]. As such, it is essentially immortal.

The naked mole rat is especially resistant to age-related physiological decline. A key reason for this may be an altered cGMP–AMP synthase (cGAS) pathway that enhances DNA repair, thereby contributing to their resistance and longevity [38]. These hairless mouse-sized rodents are native to arid regions of Africa, where they live in subterranean burrows with low oxygen levels and limited access to water. Unique mineralocorticoid receptor signalling may help regulate fluid homeostasis, contributing to their evolutionary adaptation to this arid environment [39]. To survive the hypoxic conditions, they endogenously produce fructose that, when metabolised, stimulates glycolysis to reduce oxygen requirements [40]. Fructose metabolism is associated with high mitochondrial oxidative stress and lactate formation [41], but naked mole rats generate little lactic acid under stress, reducing the toxic build-up that normally occurs in oxygen-deprived tissues. This protective mechanism results from the increased production of antioxidants, orchestrated by upregulation of Nrf2 [42]. This powerful antioxidant system may also contribute to their longevity (>30 years), lack of arterial stiffening [43], resistance to cancer [44] and insulin sensitivity [45]. Longevity in naked mole rats is further supported by high expression levels of the Klotho gene in both the liver and the kidneys, where it contributes to metabolic regulation, antioxidant defence and protection against age-related decline [46]. In addition, while the presence of hypoxia impairs insulin signalling in the naked mole rat, the queens use IGF-1 to maintain blood glucose regulation, enabling them to remain for extended periods in hypoxic nest chambers [47]. A low prevalence of end-stage renal disease and lack of common kidney diseases have also been reported in naked mole rats [48]. While protected from cancer, CVD, diabetes, kidney disease and ageing, naked mole rats are not immune to environmental threats. Surface habitat degradation, climate change that alters underground conditions, and human disturbances threaten their burrows and disrupt the delicate balance of their subterranean environment.

Bats also provide unique insights into mammalian adaptations to exposome stress. Bats are one of the largest groups of mammals on the planet, with unique physiological abilities, including powered flight and echolocation. Flying mammals have evolved to ameliorate age-related physiological decline, through superior immune resilience and enhanced DNA repair mechanisms. This also includes adapted metabolic regulation to induce torpor or hibernation [49]. The little brown bat, for example, exhibits extraordinary tolerance to environmental stress, and hibernates in freezing temperatures to survive long periods without food. While an animal’s size often correlates with its lifespan, the most interesting species are those that defy this rule. For instance, Brandt’s bat (*Myotis brandtii*), which weighs only 4–9 g, has a lifespan of 40 years, approaching that of an elephant. This bat model challenges established lifespan predictions, surviving up to ten times longer than anticipated, while demonstrating robust immune responses and a remarkable rarity of cancer [50]. Thus, bats have emerged as an unconventional mammalian system for studies of healthy ageing.

Another example of an animal that has adapted to environmental stress is the axolotl. These aquatic salamanders, native to the ancient lakes of Mexico, are renowned not only for their ability to regenerate limbs, spinal cords and even parts of their brains, but also for their resistance to cancer, wound healing and negligible senescence [51]. They remain in a larval state throughout life (neoteny), thriving in cool, oxygen-rich freshwater environments. Their regenerative ability and resistance to cancer have made them a model organism for scientific research.

Studies in elephants have also informed cancer research. Remarkably long-lived for their size, elephants typically reach 60–70 years and show a surprisingly low incidence of cancer despite having many more cells than humans because of their large body size. A key to this resilience is their multiple copies of the *TP53* gene [52]—around 20 compared with just one in humans—which produce abundant levels of p53 protein, a powerful tumour suppressor that triggers damaged cells to self-destruct. This genetic adaptation is likely to play a major role in both their longevity and cancer resistance.

The rougheye rockfish is a deep-sea dweller that is found in the North Pacific and which has a lifespan of >200 years. Living at depths of 150–450 m, rockfish endure high pressures, low temperatures and limited light. Their slow growth rate and late maturity are key traits for survival in this stable but resource-scarce environment. An analysis of the genomes of 88 rockfish species identified 137 specific genes that contributed to their longevity [53]. Two distinct networks were linked to longevity: the first involved genes related to insulin signalling and the second genes involved in the metabolism of flavonoids (found in plant-based foods) [54]. Studies such as these emphasise how genetic innovations support life-history trait adaptations and ultimately shape genomic diversity.

One of the longest-lived vertebrates is the Greenland shark, with a lifespan reaching >400 years. Inhabiting the icy depths of the Arctic and North Atlantic, these sluggish creatures survive in near-freezing darkness and have an extraordinarily slow metabolic rate. This adaptation supports both their longevity and their delayed reproduction, which may not occur until they are 150 years old. The Greenland shark has a functionally connected network enriched for DNA repair that depends on specifically duplicated genes [55].

The ocean quahog, known as the Icelandic clam, is another exceptionally long-lived species, with some individuals reaching >500 years of age [56]. Living in the cold, low-energy, low-risk environment of the North Atlantic, these clams grow slowly and maintain a low metabolic rate. They have evolved exceptional oxidative stress resistance and efficient cellular repair systems**,** which minimises cumulative damage over time. Their shells record annual growth rings, making them valuable indicators of historical oceanic and climatic conditions.

Despite the resilience of these species to extreme environments, all these species are subject to anthropogenic threats. Deep-sea fishing, bycatch, and the accumulation of heavy metals and microplastics in ocean ecosystems pose significant risks to the Greenland shark, while filter feeders, such as the quahog, are exposed to pollutants and microplastics. Thus, the rapid pace of current climate change, fishing and toxin exposures may outstrip the ability of these species to adapt.

## Insights into CVD and implications for human health

As reported in 2023, CVD has become the leading global cause of human mortality in the modern world, with an annual death toll of 18–20 million people [57]. One of the major types of CVD is cardiac hypertrophy and heart failure, especially heart failure with preserved ejection fraction (HFpEF). Often triggered by chronic hypertension, HFpEF can be associated with cardiac fibrosis and early mortality. Giraffes (*Giraffa camelopardalis*) have very high blood pressure, which is required to perfuse the brain, located 2–2.5 m above the heart, with valves in the arterial circulation helping to maintain the high pressure. Despite this massive hypertension, giraffes have a natural resistance to pressure‐induced left ventricular thickening and hypertrophy [58]. Cardiac fibrosis is minimal, and diastolic function is maintained by the renin–angiotensin and fibroblast growth factor receptor like 1 (FGFRL1) systems. Moreover, major evolutionary changes have been identified in the HOX, NOTCH (fundamental developmental signalling systems) and fibroblast growth factor (FGF) signalling pathways, which are key regulators of both skeletal morphology and cardiovascular development [59]. These adaptations shed light on the giraffe’s ‘towering genome’ [60] and suggest new strategies for managing HFpEF, a leading cause of heart failure in women.

Cardiac remodelling and hypertrophy are associated with poor outcomes in humans and can take years to reverse. Burmese pythons (*Python bivittatus*), however, have a remarkable capacity to both induce and reverse cardiac remodelling. Following a large meal, python hearts undergo rapid hypertrophy, initiating within hours and peaking within 1–2 days, driven by changes in fatty acid metabolism, and subsequently regress to baseline over the next 2–3 weeks as digestion proceeds [61]. This remodelling establishes a framework for understanding the reversibility of pathological cardiac changes in humans [62].

While CVD is the leading cause of death in humans, it is striking how rarely atherosclerosis and related conditions are observed in the animal kingdom. Investigating animal physiological adaptations related to athero-resistance and sensitivity may lead to a better understanding of disease pathophysiology and therapeutic strategies. It is difficult to estimate the relative vulnerability vs resistance to atherosclerosis in non-human animals given the small numbers of necropsies performed and lack of exposure information for affected and unaffected individuals. However, intriguing metabolic profiles found in specific taxa have sparked interest in resistance adaptations and argue for greater attention to be paid to certain species. For example, the lipid profile of seals is characterised by high levels of plasma HDL-cholesterol, normal cholesterol levels and a greater molecular size of apolipoprotein E (ApoE) compared with that of humans [63]. The last observation suggests that pinniped ApoE has a greater capacity for removal of atherogenic lipoproteins. In addition, high levels of antioxidants that are generated by cycles of ischaemia during submersion and reperfusion could, theoretically, confer athero-protection [64].

In contrast, great apes and monkeys are atherosensitive [65]. One potential explanation is that humans and apes lost uricase function because of mutations that occurred 12–15 million years ago. Notably, old-world monkeys and new-world monkeys display low levels of uricase activity [66]. They tend to have higher uric acid levels than most other mammals, and levels can rise further with diets rich in purines (such as red meats) or sugar. Soluble uric acid has been found to induce endothelial dysfunction, activate thromboxane and stimulate reactive oxygen species (ROS) production and the renin–angiotensin–aldosterone (RAAS) system [67]. In addition, urate crystals have been localised to atherosclerotic plaques in humans with gout, where they may stimulate local inflammation. Coupled with Western diets and inadequate exercise from inappropriate keeping conditions, this make apes susceptible to disease [68]. In contrast, prosimians with a functioning uricase gene rarely develop atherosclerosis which may be related to their low uric acid levels and high vitamin C levels [69], which neutralise ROS [66]. Birds, like apes, are also highly susceptible to atherosclerosis, particularly when exposed to captivity-related risk factors and comorbidities such as obesity, hyperlipidaemia, lack of exercise, ageing, and cardiovascular, hepatic and reproductive diseases [70]. The high sensitivity of birds to atherosclerosis may in part be due to their lack of uricase and high uric acid levels, as well as lack of ApoE [71] and many of the major proteins involved in cholesterol internalisation [72].

## Harnessing hibernation for the treatment of metabolic disorders

Hibernation represents the ultimate survival strategy when food and water are unavailable, and predators need to be avoided. The decrease in metabolic rate during hibernation is followed by a drop in body temperature and evaporative water loss, allowing animals to obtain both water and energy from the slow oxidation of fat. In small animals, such as the ground squirrel, body temperature may reach 4°C or lower accompanied by extreme bradycardia (4–5 bpm in ground squirrels). Periods of torpor, representing transient reductions in metabolism to conserve energy, are periodically interrupted by brief arousal episodes of 24–48 h. Favourable metabolic changes during hibernation slow down epigenetic ageing in both big brown bats [73] and marmots [74].

**Fig. 3:**
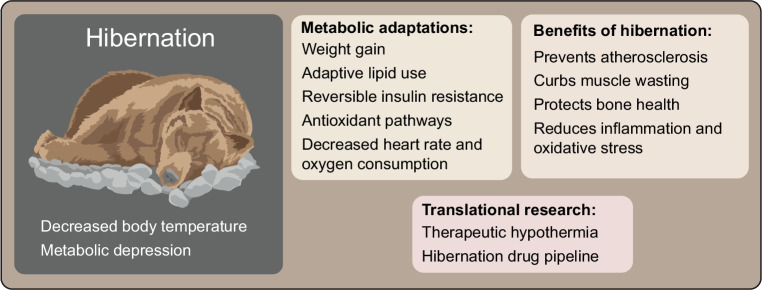
Hibernation provides a unique physiological model for developing therapeutic strategies against metabolic and degenerative diseases. During hibernation, mammals undergo a tightly regulated state of metabolic depression characterised by profound reductions in body temperature, heart rate and oxygen consumption. Despite extended fasting and immobility, hibernators maintain stable glucose homeostasis, insulin sensitivity and preservation of lean tissue mass. These effects are mediated through reversible insulin resistance, adaptive lipid use and suppression of systemic inflammation. Translational research is exploring pharmacological agents that mimic hibernation-like states with the aim of modifying metabolism and improving health. This figure is available as part of a downloadable slideset

Much can be learned from hibernation. Animals have found ways to prevent atherosclerosis during hibernation despite demonstrating insulin resistance and marked hyperlipidaemia, as well as muscle wasting and bone loss from disuse (Fig. 3). For example, in hibernating bears, energy loss in skeletal muscle is limited by reducing myosin ATPase activity [75]. Additionally, hibernating animals store phosphocreatine in their brains, hearts and skeletal muscle, which can be used as a substrate for creatine phosphokinases to generate an alternative source of ATP to satisfy energy needs. While brown adipose tissue can generate heat when oxidised, skeletal muscle plays a key role in non-shivering thermogenesis in hibernators, especially during arousal phases. This is especially important because of the significant energy required to come out of hibernation. The resistance of hibernating bears to atherosclerosis despite having highly elevated plasma lipid levels [76] may be due to a natural immunisation against atherosclerosis [77] and the vasculo-protective properties of plasma lipoproteins [78]. Hibernation is also associated with protection against oxidative stress, which also plays a role in metabolic disease [79]. This appears to be due to decreased levels of proinflammatory cytokines [80] and increased expression of Nrf2 [81]. We observed in free-ranging brown bears that choline is redirected to produce massive amounts of betaine during hibernation [82]. As betaine is a methyl donor that acts as an osmoprotectant, which safeguards protein function and serves as an exercise mimetic [83], its potent multi-organ geroprotective effects warrant further exploration for the treatment and prevention of non-communicable diseases.

Hibernation is also important for understanding weight gain and what causes the switch to low-grade fat metabolism, reduced energy expenditure, insulin sensitivity modulation and lipid use, all of which are tightly regulated to maintain metabolic homeostasis. Understanding these adaptations could inspire novel therapeutic strategies for obesity, type 2 diabetes, atherosclerosis, osteoporosis, muscle loss and metabolic diseases [79]. One remarkable feature of hibernation is the reversible insulin resistance [8]. The dynamic control of insulin resistance in bears contrasts with the persistent insulin resistance seen in human obesity and type 2 diabetes. Research into the molecular pathways regulating reversible insulin resistance has identified roles for adipokines, AMP-activated protein kinase (AMPK) and peroxisome proliferator-activated receptors (PPARs), all of which are potential drug targets for metabolic diseases [84]. Translational research is exploring pharmacological agents that mimic hibernation-like states in a ‘hibernation drug pipeline’ [85], including torpor-inducing compounds or metabolic suppressants that lower energy expenditure. It has even been questioned whether human hibernation may be a strategy for extended space travel. Indeed, evidence suggests that our distant evolutionary ancestors (hominins) may have mastered the trait of hibernation [86].

## Lessons from resilient species on adapting to global environmental change

**Fig. 4:**
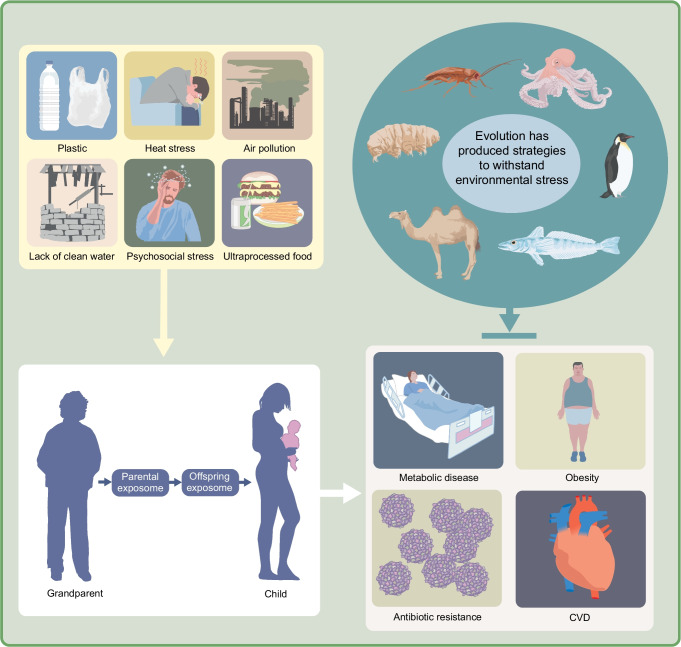
Natural models of resilience: lessons from evolution for environmental health and longevity. Environmental exposures can leave epigenetic marks that influence the health of future generations, creating intergenerational and transgenerational ripple effects. Intergenerational inheritance reflects direct physiological exposures, whereas transgenerational inheritance involves stable transmission of epigenetic information across unexposed generations, a deeper form of biological memory. Nature provides multiple examples of how evolution has produced diverse strategies to withstand environmental stress. Camels regulate their body temperature and conserve water through specialised blood cell morphology, illustrating strategies for heat tolerance and hydration. Killifish enter suspended animation during droughts and have adapted to toxic waters, providing a model for metabolic slowdown, hypoxia tolerance and pollution resistance. Tardigrades survive total desiccation and radiation through vitrification and protective proteins, informing approaches to tissue preservation and radioprotection. Cockroaches demonstrate robust detoxification and DNA repair capacity, revealing mechanisms of cellular stress resistance. Penguins maintain efficient oxygen use and lipid metabolism during prolonged dives and fasting, exemplifying adaptation to hypoxia and energy scarcity. Octopuses exhibit temperature-dependent RNA editing, providing a model for molecular flexibility and rapid environmental adaptation. Studying such mechanisms may yield translational insights for enhancing human resilience to climate-related and environmental health challenges. Solutions inspired by nature may also provide novel insights to help us combat the growing burden of lifestyle-related diseases. This figure is available as part of a downloadable slideset

The impact of anthropogenic damage to planetary health, particularly climate change and habitat loss, will be felt across generations in all constituents of the biosphere. Lifelong exposure to the exposome influences health and ageing trajectories by dynamically modulating epigenetic responses to environmental change [1]. As environmental changes accelerate ageing, ignoring planetary health represents a critical gap in geroscientific research [87]. Changes in the environment can influence fetal development and health trajectories in subsequent generations. For example, a parent’s exposome can influence their offspring’s ageing and health, while a grandparent’s exposome (typically via nutrition and exposure to biopsychosocial stresses) can affect parental sperm and egg physiology; hence, the impact is felt generations later [88] (Fig. 4). Indeed, such effects are still detectable up to six generations later [89], highlighting the need to mitigate these negative outcomes. Thus, biomimetic studies of resilient species are of critical and immediate importance if humanity is to adapt and enhance its physiological resilience in the face of man-made crises [27].

Environmental stress, including temperature extremes, pollution, water scarcity, hypoxia, habitat degradation and loss of biodiversity, affects all life forms. Some species, however, exhibit extraordinary resilience and longevity, offering insights into how biology can adapt to harsh or changing conditions [36]. Among species that have evolved unique resilience to environmental stress are the wood frog (*Lithobates sylvaticus*) (survives winter by freezing solid), killifish (Cyprinodontiformes) (has developed evolutionary resistance to pollution), tardigrades (Tardigrada) (employ cryptobiosis to protect DNA), Antarctic icefish (*Channichthys rhinoceratus*) (produces antifreeze glycoproteins to prevent ice crystal formation in subzero waters), octopus (Octopoda) (undergoes temperature-dependent RNA editing) and cockroaches (demonstrate how environmental resilience intersects with microbial adaptation) [90]. Each of these species has evolved unique strategies that also reveal their hidden vulnerabilities in the face of global environmental change.

Microplastic exposure is also now a pervasive global issue, with estimates suggesting that humans ingest about 5 g per week, roughly the weight of a credit card. With an estimated 350,000 cases of CVD reported to be attributable to plastics annually, the magnitude of this emerging health threat is far greater than expected [91]. While some animals have developed remarkable adaptations to cope with short-term plastic exposure [92], we must learn more from them to understand potential resilience mechanisms. The bidirectional relationship between microplastics and gut microbiota has significant implications for animal and human health [93].

## Summary: resilience, longevity and metabolic adaptation in extreme animal phenotypes

Many animal species have evolved remarkable adaptations that enable survival under conditions that are often considered extreme. These adaptations primarily support access to essential resources such as energy, water and oxygen and ensure safe and effective reproduction. Such resilience is often linked with healthy longevity and a low risk of disease. Studying these evolutionary strategies not only enhances our scientific understanding but could also inform the development of future therapies.

Nevertheless, climate change and human exploitation are imperilling even well-adapted species [3]. Scientists warn that a sixth mass extinction is underway, driven primarily by human activities including habitat destruction, climate change and pollution [94]. Eliminating entire branches of the tree of life threatens the unique evolutionary history embodied in each one that is lost. Many animals hold valuable insights for preventing and treating human diseases. While life continuously evolves into new possibilities within its interconnected ecosystem, safeguarding biodiversity is essential for securing a healthy future for humanity.

## Supplementary Information

Below is the link to the electronic supplementary material.

Slideset of figures (PPTX 822 KB)

## Abbreviations

ApoE: Apolipoprotein E
Bpm: Beats per minute
GLP-1: Glucagon-like peptide-1
HFpEF: Heart failure with preserved ejection fraction
Nrf2: Nuclear factor erythroid 2-related factor 2
ROS: Reactive oxygen species

## Authors’ relationships and activities

RJJ is on the scientific boards of Santa Barbara Nutrients, RxSugar and A New Dimension and has equity with Colorado Research Partners LLC. PGS sits on the advisory board of AKL Therapeutics, the Danone Institute and A New Dimension. PS has participated on scientific advisory boards for CSL, Novo Nordisk, AstraZeneca, Baxter, Fresenius Medical Care, Invizius and Vera. PK is a former employee of the Renal Research Institute, a wholly owned subsidiary of Fresenius Medical Care. PK also holds stock in Fresenius Medical Care and is inventor on multiple patents in the kidney space. The authors declare that there are no other relationships or activities that might bias, or be perceived to bias, their work.
