# Supplementary material for: Comparative physiology and biomimetics in metabolic and environmental health: what can we learn from extreme animal phenotypes?
Source: Diabetologia. 2025 Nov 20;69(2):295–307. doi: 10.1007/s00125-025-06611-3 (PMC12779663; doi:10.1007/s00125-025-06611-3)
Supplement: Supplementary file 1 — Slideset of figures (PPTX 822 KB) [file 125_2025_6611_MOESM1_ESM.pptx]

## Slide 1
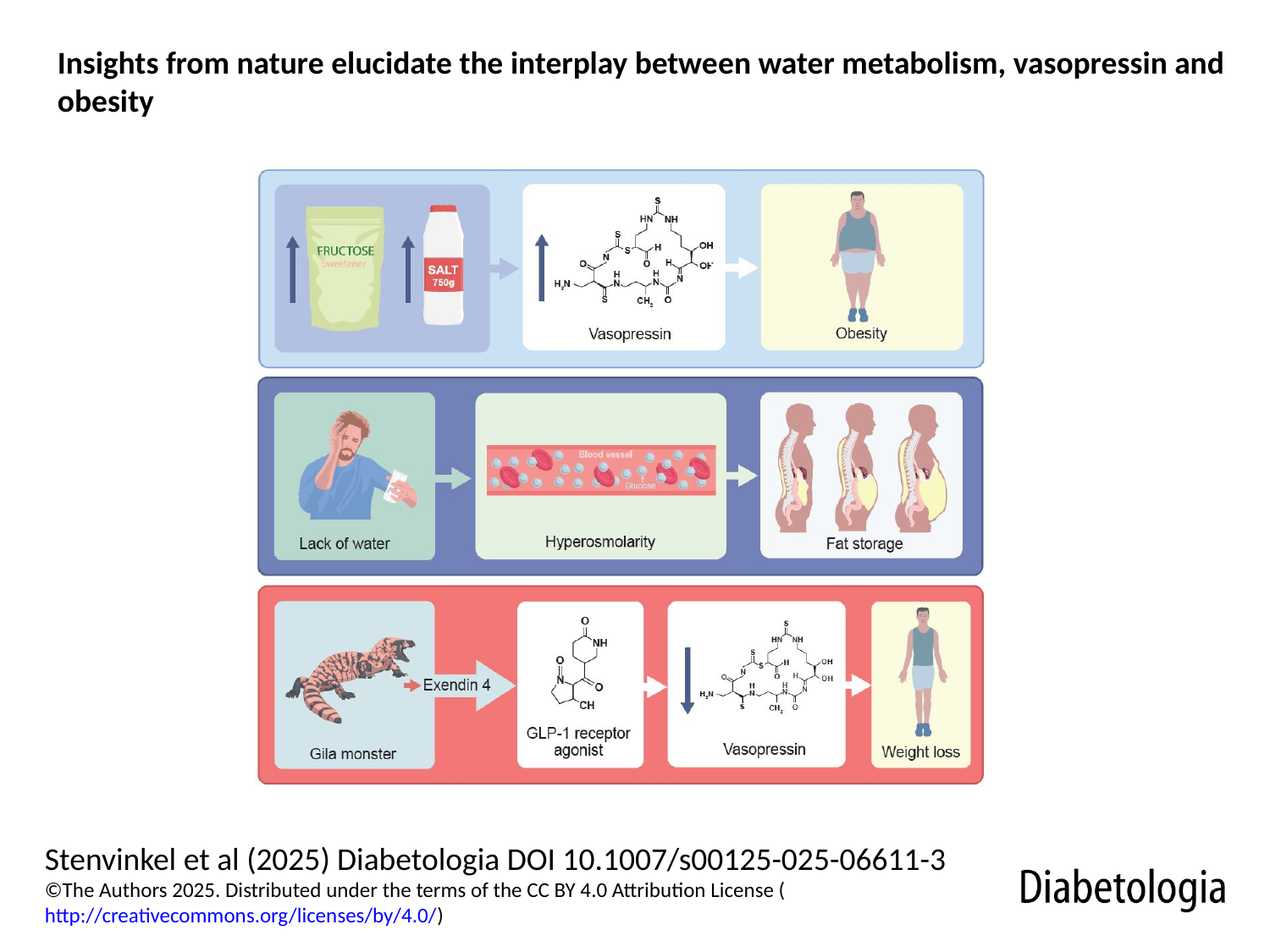

Insights from nature elucidate the interplay between water metabolism, vasopressin and obesity
Stenvinkel et al (2025) Diabetologia DOI 10.1007/s00125-025-06611-3
©The Authors 2025. Distributed under the terms of the CC BY 4.0 Attribution License (http://creativecommons.org/licenses/by/4.0/)

## Slide 2
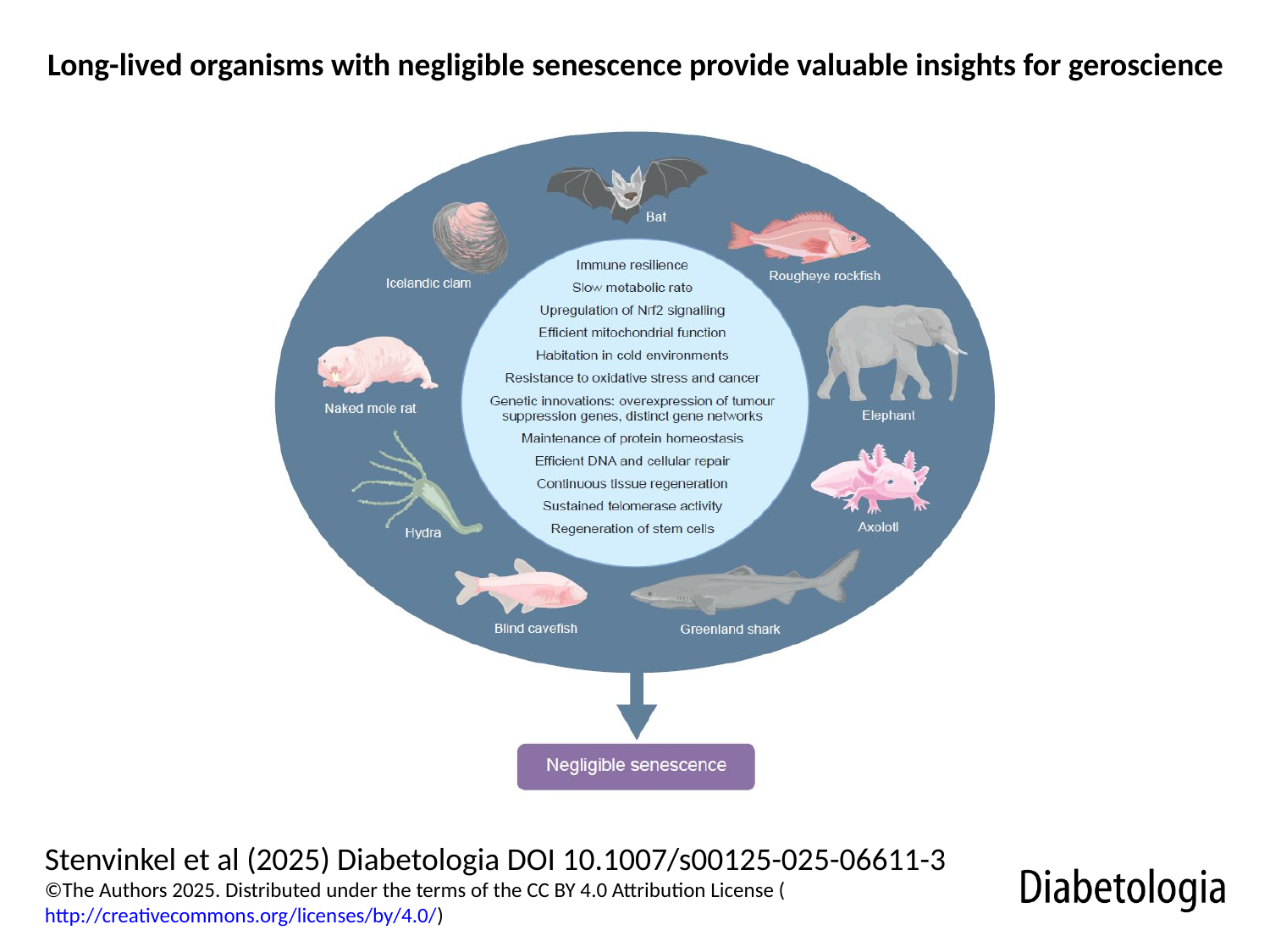

Long-lived organisms with negligible senescence provide valuable insights for geroscience
Stenvinkel et al (2025) Diabetologia DOI 10.1007/s00125-025-06611-3
©The Authors 2025. Distributed under the terms of the CC BY 4.0 Attribution License (http://creativecommons.org/licenses/by/4.0/)

## Slide 3
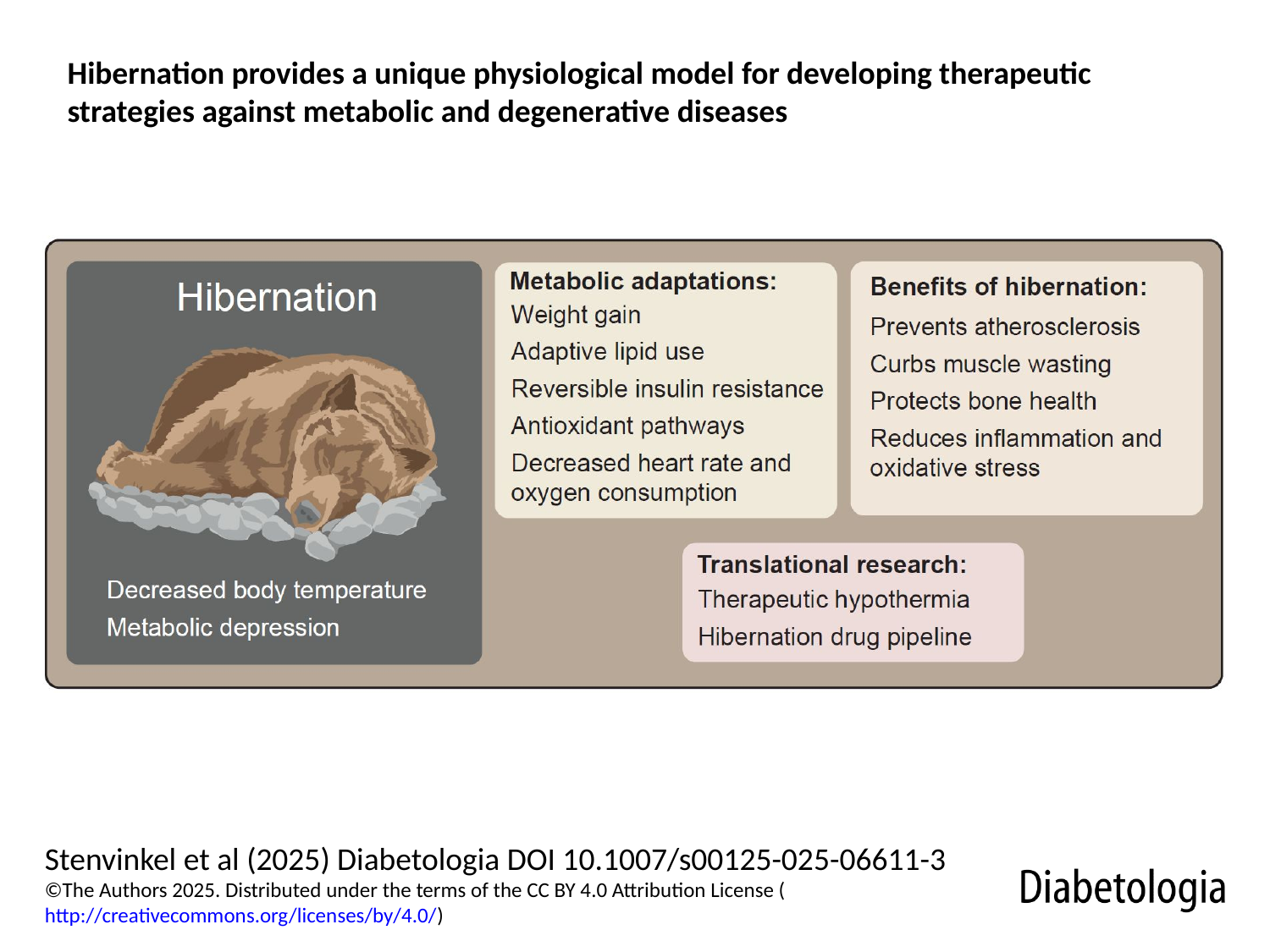

Hibernation provides a unique physiological model for developing therapeutic strategies against metabolic and degenerative diseases
Stenvinkel et al (2025) Diabetologia DOI 10.1007/s00125-025-06611-3
©The Authors 2025. Distributed under the terms of the CC BY 4.0 Attribution License (http://creativecommons.org/licenses/by/4.0/)

## Slide 4
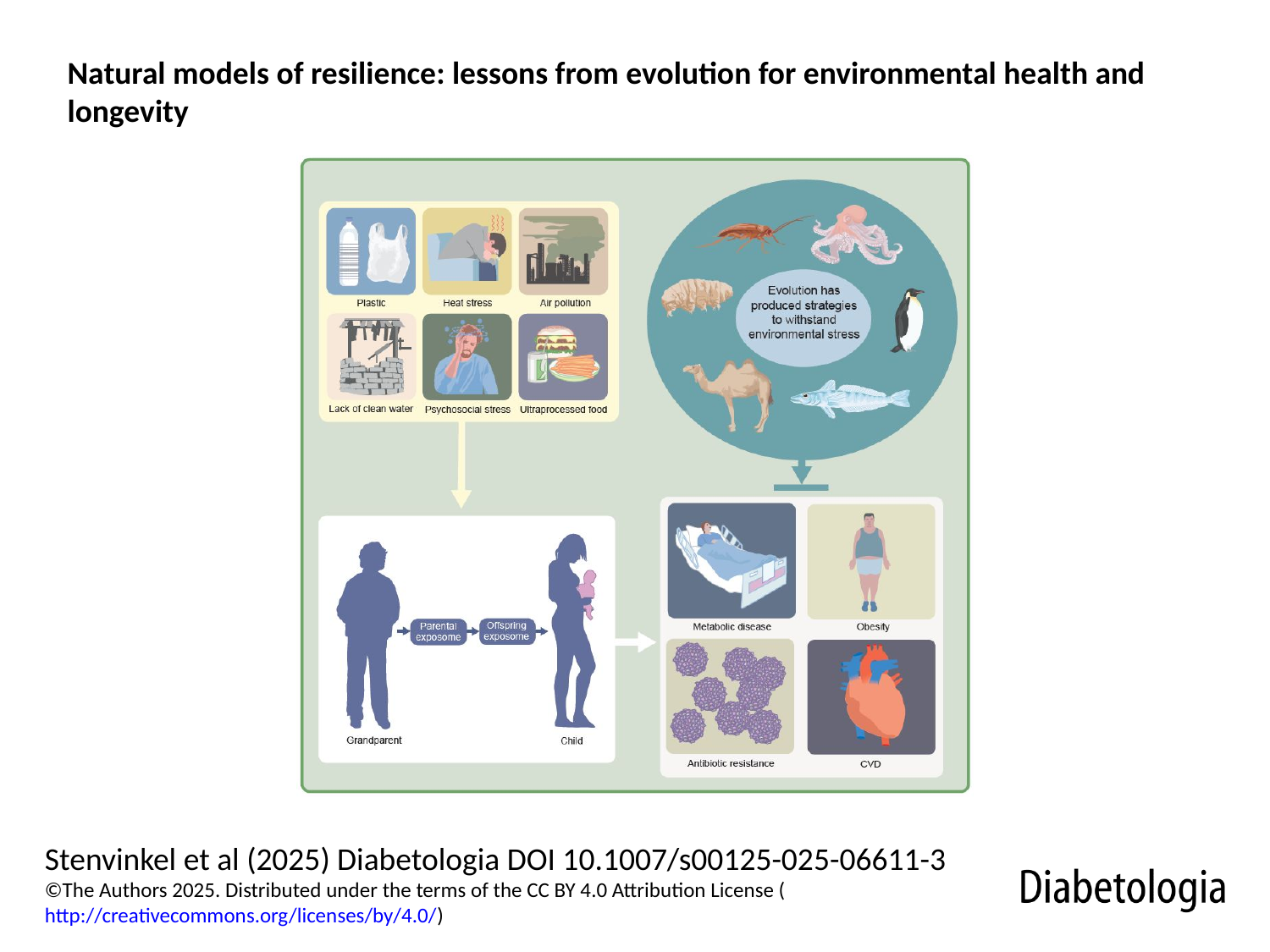

Natural models of resilience: lessons from evolution for environmental health and longevity
Stenvinkel et al (2025) Diabetologia DOI 10.1007/s00125-025-06611-3
©The Authors 2025. Distributed under the terms of the CC BY 4.0 Attribution License (http://creativecommons.org/licenses/by/4.0/)
